# Perspectives on oral pre-exposure prophylaxis use amongst female sex workers in Harare, Zimbabwe

**DOI:** 10.4102/sajhivmed.v21i1.1039

**Published:** 2020-02-19

**Authors:** Tinashe Mudzviti, Anesu Dhliwayo, Byrone Chingombe, Bernard Ngara, Tsitsi G. Monera-Penduka, Charles C. Maponga, Gene D. Morse

**Affiliations:** 1 School of Pharmacy, University of Zimbabwe, Harare, Zimbabwe; 2 Newlands Clinic, Harare, Zimbabwe; 3 Population Services International, Harare, Zimbabwe; 4 Department of Community Medicine, College of Health Sciences, University of Zimbabwe, Harare, Zimbabwe; 5 Center for Integrated Global Biomedical Sciences, University at Buffalo, New York, United States; 6 Translational Pharmacology Research Core, University at Buffalo, New York, United States

**Keywords:** female sex workers, HIV, pre-exposure prophylaxis, barriers, Truvada

## Abstract

**Background:**

Pre-exposure prophylaxis (PrEP) could provide protection from human immunodeficiency virus (HIV) infection in sexually active persons at risk. Limited data are available in Zimbabwe with regard to the perceptions about PrEP amongst female sex workers (FSWs).

**Objectives:**

The aim of this study was to evaluate the knowledge levels of oral PrEP and the likelihood of its use amongst FSWs.

**Method:**

This was a cross-sectional study in the peri-urban areas of Harare, Zimbabwe. Human immunodeficiency virus-negative FSWs were interviewed to assess their awareness of and likelihood to use PrEP. The relative importance index was used to evaluate the levels of knowledge and the likelihood of, and barriers to, PrEP use. A set of 10 questions was designed and validated that evaluated participants’ understanding of PrEP. A bivariate logistic regression model was utilised to identify predictors of PrEP use.

**Results:**

A total of 131 FSWs with a median age of 25 years (interquartile range: 21–31) participated in this study. Of the 71 (54%) FSWs who had heard about PrEP, 46 (35%) participants had adequate knowledge of its use. A total of 102 (78%) participants revealed that they would be willing to continuously use PrEP if it was provided free of cost. Increasing age of the participants was associated with an increase in the likelihood of using PrEP (*r* = 0.0033, *p* = 0.038). More knowledge about PrEP increased the likelihood of its use (*r* = 0.21, *p* = 0.0153). This likelihood increased amongst participants with an unprotected sexual intercourse encounter in the preceding 3 months (*r* = 0.0448, *p* = 0.026).

**Conclusion:**

Knowledge of PrEP amongst FSWs was low. To increase the uptake of PrEP, there is a need to further sensitise FSWs about this intervention. Programmes should also promote awareness training in FSW subgroups that are less likely to use PrEP.

## Background

Whilst the adult prevalence of human immunodeficiency virus (HIV) in the general population of Zimbabwe is 15%, the prevalence in key populations is higher.^1^ In populations of female sex workers (FSWs), the HIV prevalence in 2013 was 50% – 70% in different parts of Zimbabwe.^2^ In many settings, key populations are hidden and stigmatised, and their representation in national surveillance data is limited. It has been cited that key populations and their sex partners not only make up the largest proportion of people living with HIV (PLWH) but also represent a significant proportion of new infections in sub-Saharan Africa.^3^

Pre-exposure prophylaxis (PrEP) can become a female-controlled HIV prevention method for FSWs and others who are unable to negotiate condom use. The Ministry of Health and Child Care (MoHCC) has developed a PrEP framework policy that prioritises access for ‘at-risk’ populations. Groups that need to be offered PrEP include female and male sex workers; serodiscordant couples, that is, the HIV seronegative partner; adolescent girls and young women; pregnant women in relationships with men of unknown status and high-risk men, for example, men who have sex with men (MSM); prisoners; long-distance truck drivers; and transgender people.^4^ A fixed-dose regimen of either Tenofovir 300 mg and Emtricitabine 200 mg (TDF/FTC) or Tenofovir 300 mg and Lamivudine 300 mg (TDF/3TC) has been recommended for once-daily oral administration during the period an individual is at risk of contracting HIV infection.^4^ Zimbabwe has adopted The Joint United Nations Programme on HIV and AIDS 90-90-90 global goals to help reduce new infections and end the HIV pandemic.^5^ This target will be difficult to reach without reducing transmission amongst high-incidence populations.

Although it is well established that effective PrEP could provide an additional safety net to sexually active persons at risk,^6^ limited data are available in Zimbabwe regarding the knowledge and the likelihood of PrEP use amongst FSWs. There is a need to understand both the acceptability of PrEP amongst FSWs and the factors likely to determine uptake. Most research efforts to date have focussed on clinical aspects of PrEP. Little attention has been focussed on the factors that influence FSWs’ willingness to take it. It is estimated that there are 40 000 (plausibility bounds [PBs] 28 000–59 000) active FSWs in Zimbabwe, that is, 1.23% (PB: 0.86% – 1.79%) of the adult female population. A total of 20 000 (50%) are in Harare and Bulawayo.^7^ This study was conducted to assess the levels of knowledge, barriers to and likelihood of oral PrEP use amongst FSWs as a preventative method in reducing the risk of acquiring HIV in Harare, Zimbabwe.

## Methods

### Study design and setting

This was a cross-sectional study of FSWs in seven peri-urban areas of Harare province, Zimbabwe. These sites were specifically chosen because they are high-density areas, and apart from the central business district, they are areas from which FSWs frequently operate.^8^

### Study population and recruitment

This cross-sectional study was conducted between December 2016 and February 2017 in partnership with a local private voluntary organisation (PVO) that offers PrEP services. The PVO had been offering PrEP using TDF/FTC (Truvada^TM^) tablets as an HIV prevention method in six Zimbabwean districts since August 2016. This was a demonstration project introduced to inform people of the new HIV prevention strategy of the MoHCC. The primary target populations included adolescent girls and young women aged 15–24 years, FSWs, MSM and serodiscordant couples.^9^

Human immunodeficiency virus–negative FSWs were defined as those who had been tested for HIV in the previous 3 months and had tested negative or those who perceived themselves to be HIV-negative. The PrEP intervention that is defined for the purpose of this study refers to oral PrEP with Truvada, which is indicated for ‘at-risk’ individuals with a laboratory-confirmed HIV-negative result. None of the participants was already receiving PrEP from the PVO.

Snowball sampling was used to locate and enrol FSWs from the peri-urban Harare sites. A peer referral system whereby ‘seed’ subjects previously identified by the PVO and ‘queens’ (the leaders of a significant group of sex workers based on their location in a certain area) provided referrals to enable further recruitment and mobilisation of other FSWs. This technique was utilised because of laws and policies that criminalise sex work in Zimbabwe. Female sex workers were also identified during community outreach HIV testing and counselling activities by the PVO. Once identified, FSWs were asked to provide written consent and to complete a 48-item questionnaire. The questionnaire was administered by the interviewer. The questionnaire had sections enquiring about socio-demographic factors, sexual behavioural characteristics, HIV testing, PrEP knowledge, perceptions, barriers and the likelihood of its use. Sexual behavioural characteristics were investigated to determine the HIV acquisition risk profile of the FSWs. The interviews were conducted in the language that the participant was most comfortable with. ‘Sufficient knowledge’ was judged by means of an initial self-report and an additional nine technical questions designed to test the level of knowledge. These technical questions included the participant’s knowledge that PrEP is used by HIV-negative individuals, the dosing frequency, potential drug interactions and whether there are other reproductive health benefits. Once the level of knowledge was ascertained, the participants were then educated about PrEP use. The perceived barriers that were evaluated included stigma, cost of PrEP and the side-effects of Truvada.

### Data management and statistical analysis

Research Electronic Data Capture (REDCap) was used to manage data. This was hosted by the College of Health Sciences of the University of Zimbabwe. The contribution of each factor, namely, age, cost of PrEP and drug side-effects, with regard to improving PrEP uptake amongst FSWs was examined. The importance of each factor as perceived by the respondents was assessed by computing the relative importance index (RII). The RII is a statistical measure recorded on a scale of 0 < RII ≤ 1, where ‘0’ or any value close to ‘0’ is defined as a poor knowledge of PrEP use, the participant is less likely to use PrEP or barriers associated with PrEP are likely to affect uptake. If the RII score was ‘1’ or close to ‘1’, it means that participants were knowledgeable about PrEP, were more likely to use PrEP and that barriers associated with PrEP use were unlikely to affect its uptake.

The RII was computed using the equation $\text{RII}=\sum \frac{Scorei}{Kn}\times 100\%$, where Score_i_ was the score for each question given by the participants and ranged from 0 to 4 (where ‘0’ was ‘very much disagree, strongly disagree or never’ and ‘4’ was ‘strongly agree, very much agree or almost always’). *K* was the maximum possible score and *n* was the total number of questions. According to Johnson and LeBreton,^10^ RII is the proportionate contribution each predictor makes to *R*^2^ (where *R*^2^ is the extent to which the dependent variable can be predicted by the predictor variables), considering both its direct effect (correlation with the dependent variable) and its effect when combined with other variables.

All data analysis was performed using Stata version 13 (StataCorp LP, TX, USA) software package. Bivariate linear regression was used to determine if there was a relationship between the RII scores of knowledge, likelihood and barriers (KLBs) and exploratory factor variables that were collected in the study. To identify the relationship between KLB RII scores, a matrix of Spearman correlation coefficients was used.

### Ethical consideration

Prior to conducting the survey, the study protocol including the data collecting tool was reviewed and approved by the Joint Research Ethics Committee of the University of Zimbabwe and the Parirenyatwa Group of Hospitals (JREC/328/16). All participants provided written informed consent before participating in the study.

## Results

A total of 131 presumed HIV-negative, adult FSWs were recruited to participate in this study, and their demographic characteristics are shown in Table 1. All study participants were self-identified as residents of the Harare province.

**TABLE 1:** Demographic characteristics of study participants (*N* = 131).

Variable	Outcome	
	*N*	%
Age (years), median (IQR)	25 (21–31)	-
**Marital status**		
Single	102	78
Married	14	10
Divorced	15	11
Years in practice as a sex worker, median (IQR)	2 (1–4)	-

IQR, interquartile range.

### Participant’s characteristics – Sexual risk factors and human immunodeficiency virus testing

The median number of sexual encounters by the participants was five [interquartile range (IQR): 3–6] partners per day. Half of the participants (50%) did not know their partner’s HIV serostatus and only 42% of the FSWs would talk about HIV with their clients or partners. All participants perceived the use of condoms as a necessary tool when engaging in sexual activity with their partners or clients, 86% used condoms with the last three partners they encountered and 44% reported having ever had a condom burst at least once during sexual intercourse. The variables affecting HIV acquisition risk are depicted in Table 2.

**TABLE 2:** Variables affecting risk of human immunodeficiency virus acquisition.

Variable	Outcome	
	*N*	%
**Number of partners encountered a day, median (IQR)**	5 (3–6)	-
**Any unprotected sex in the last 3 months**		
YES	63	48
NO	68	52
**If YES, was this with a casual partner**		
Always	43	68
Sometimes	10	16
Never	10	16
**Use of condom with the last three partners**		
Always	112	86
Sometimes	13	10
Never	6	4
**Instances where condom burst**		
YES	58	44
NO	73	56
**Talk about HIV with partner(s)**		
Always	55	42
Sometimes	32	24
Never	44	34

IQR, interquartile range; HIV, human immunodeficiency virus.

### Pre-exposure prophylaxis knowledge

Of the 131 participants, 71 (54%) had heard about PrEP and of those only 46 (35%) had sufficient knowledge about PrEP (RII > 0.5). Participants mostly heard about PrEP from non-governmental organisations (59%), from friends (35%) and only 6% of the participants had heard about PrEP through clinics.

### Likelihood of pre-exposure prophylaxis use

The participants’ responses when asked about the likelihood of PrEP use are shown in Table 3. Regardless of a likelihood to use PrEP, potential barriers were cited, such as stigma, costs, side-effects associated with the PrEP tablet and poor knowledge, as shown in Table 3.

**TABLE 3:** Likelihood of pre-exposure prophylaxis use and perceived barriers (*N* = 131).

Variable	Frequency	
	*n*	%
**Likelihood of PrEP use if provided for free**		
Sometimes	29	22
Always	102	78
**Likelihood of PrEP use if it caused mild side-effects**		
Sometimes	50	38
Always	81	62
**Condom use if FSWs were to start taking PrEP**		
Never	6	5
Sometimes	24	18
Always	101	77
**Likelihood of PrEP use if it had to be paid for**		
Never	3	2
Sometimes	41	32
Always	87	66
**Barriers associated with PrEP uptake amongst FSWs (*N* = 131)**		
**Stigma**		
Strongly disagree	128	98
Strongly agree	3	2
**Costs**		
Strongly disagree	70	53
Neutral	1	1
Strongly agree	60	46
**Side-effects associated with PrEP pill**		
Strongly disagree	117	89
Neutral	2	2
Strongly agree	12	9
**Poor knowledge**		
Strongly disagree	110	84
Neutral	1	1
Strongly agree	20	15

FSW, female sex worker; PrEP, pre-exposure prophylaxis.

### Relative importance index

On a scale of RII, the median score for PrEP knowledge was ‘0’, indicating that participants had limited knowledge about PrEP. Participants perceived the use of PrEP as an important component in the ideal HIV prevention strategy once educated about it. In relation to the likelihood of PrEP use, the median score for the likelihood of PrEP use was 0.89 ranging from 0.48 to 1. Therefore, the likelihood of PrEP use amongst the participants was high. The RII median score for barriers associated with PrEP use was 0.29 (IQR: 0–0.63). This indicated that the barriers associated with PrEP uptake were less likely than knowledge to affect participants’ use of PrEP. Results that were statistically significant in the bivariate analysis are shown in Table 4.

**TABLE 4:** Relationship between predictor variables and dependent variable.

Predictor variable	Regression parameter	*p*	Dependant variable
Dependents	−0.0636	0.020	Knowledge
Age	0.0033	0.038	Likelihood
Unprotected sex in the last 3 months	0.0448	0.026	Likelihood

An increase in the number of dependents was associated with a reduced knowledge about PrEP, as shown in Table 4. There was a statistically significant association between age and likelihood of PrEP use. As participants became older, there was an increase in the likelihood of PrEP use. This increased amongst participants who had unprotected sex in the last 3 months.

There was, however, no statistically significant association between knowledge RII with age, marital status, education, change in place of residence, income and years of practice as a sex worker (*p* > 0.05).

Considering the likelihood of PrEP use amongst the participants, there was no association noted between the likelihood RII score and the number of dependents, marital status, education, change in place of residence, income and years of practice as a sex worker (*p* > 0.05). Spearman’s rank correlation coefficient was computed to identify and test the strength of the relationship between knowledge and barriers with the likelihood of PrEP use. Table 5 shows the Spearman correlation coefficients for the relationship between RII scores of KLBs associated with PrEP use.

**TABLE 5:** Spearman correlation coefficients for the relationship between relative importance index scores of knowledge, likelihood and barriers associated with pre-exposure prophylaxis use.

Variables	Knowledge	Likelihood	Barriers
Knowledge RII	1.0000	-	-
Likelihood RII	0.2115 (*p* = 0.0153)	1.0000	-
Barriers RII	−0.0530 (*p* = 0.5476)	−0.2329 (*p* = 0.0074)	1.0000

RII, relative importance index.

Considering likelihood and knowledge RII scores, there was a positive correlation whereby more knowledge about PrEP moderately increased the likelihood of PrEP use (*r* = 0.21, *p* = 0.0153). There was a negative correlation between barriers and the likelihood RII score, that is, as barriers associated with PrEP use decreased, the likelihood of PrEP use moderately increased (*r* = 0.23, *p* = 0.0074).

## Discussion

In a real-world setting, the effectiveness of PrEP will depend on its acceptability, adoption and sustained use by high-risk populations. Pre-exposure prophylaxis medication will have little impact in reducing HIV infections if these components are not addressed.^11^ Findings from this study indicate that FSWs continue to engage in risky sexual behaviours. In the last 3 months, 53 (40%) of the participants reported having unprotected sexual intercourse with a casual partner.

In our study, 54% of FSWs had heard of PrEP before participating. In spite of having little knowledge of PrEP, the majority of FSWs were willing to use PrEP (median RII = 0.89; range 0.48–1) to reduce their risk of contracting HIV infection. In a similar study conducted in China, only 16.5% had heard of PrEP before participation, and only 1.4% had used PrEP before.^12^ Nevertheless, 69% and 95% of the respective FSW populations of China and India reported a willingness to use PrEP.^12,13^ These estimates were consistent with our findings that 89% of the participants were willing to use PrEP. Educating HIV-uninfected FSWs about PrEP is likely to support the uptake of PrEP and assist in decreasing the incidence of HIV in Zimbabwe. Pre-exposure prophylaxis programmes have been incorporated into sexually transmitted disease clinics, reproductive health programmes and genitourinary medicine clinics in high-income countries.^14,15,16,17^

Findings from this study indicated that there was a significant association between the likelihood of PrEP use, age and unprotected sexual intercourse in the preceding 3 months. Older participants were more likely to adopt PrEP as an HIV prevention strategy. This suggests that those who perceive themselves to be at a higher risk of HIV infection are more likely to adopt PrEP as an HIV intervention. Elmes et al. evaluated condom use by FSWs in Eastern Zimbabwe and reported that older participants were less likely to request condom use from partners.^18^ Older FSWs may therefore be at a higher risk of acquiring HIV and should be prioritised for PrEP access.

In spite of the high levels of interest in PrEP, potential barriers were cited including cost, side-effects and poor knowledge of PrEP use. Whilst PrEP had to be bought, 46% of the participants strongly agreed that lack of money would pose a challenge to PrEP uptake. Many participants felt that PrEP ought to be provided free in view of the severity of the HIV epidemic in Zimbabwe. The high cost of PrEP is undoubtedly a major barrier to its uptake worldwide, particularly in high-income countries where only branded TDF/FTC is available.^19^ In low-income countries that have established PrEP programmes, cost is not identified as a barrier to access because the medicines are free. In the Kenyan setting, FSWs were more concerned about the stigma of being tested for HIV and the barrier posed by the test with regard to the success of the intervention.^20^ In our study, stigma was not identified as a possible barrier to taking PrEP. This contrasted with the Kenyan setting and is a distinct finding for the Zimbabwean FSWs. With a reduced fear of stigmatisation, there is scope for improved acceptability of PrEP in FSWs.

Our results indicate that participants were generally willing to accept PrEP and adopt it as soon as it was made available. Information emerging from trial projects and open-label extensions where PrEP has been offered free of charge by knowledgeable providers suggests that uptake may be high in settings where cost and provider-related barriers had been removed.^15,16,21^

Our results showed that participants were willing to take PrEP even when reminded of potential side-effects. Participants cited that the side-effects of PrEP could have an impact on one’s quality of life but that would not hinder taking PrEP. Only 9% of the FSWs strongly expressed that side-effects would affect their lifestyle and possibly prevent PrEP use. Sex workers in Mombasa, Kenya, voiced concerns about the potential negative side-effects of PrEP. In this qualitative study, a minority of participants were deterred from using PrEP because of the side-effects.^22^ Women from six US cities where female HIV infection is highly prevalent viewed PrEP as an important prevention option, provided that side-effects and cost to the consumer were minimal.^23^

The feasibility of oral PrEP implementation in Zimbabwe has been proven in ongoing and completed demonstration projects and clinical trials. By the end of 2017, a total of 3073 clients were initiated on PrEP in Zimbabwe. Ninety per cent of the clients initiated on PrEP were women, with the majority of them in the 25–49 years age group. The majority (52%) of the clients initiated on PrEP were FSWs. The accessibility of PrEP outside of demonstration projects has been limited.^9^

Whilst the sample size recruited for participation in this study was small, the information generated provides a foundation in the development of further programming for PrEP implementation in FSWs. The information was also generated from peri-urban, high-density areas in Harare (the capital city of Zimbabwe). Perceptions and knowledge levels might differ across other diverse geographical locations in Zimbabwe, and more studies need to be conducted. Because of the legal status of sex work in Zimbabwe, we used a snowball sampling technique in order to reach FSWs who might otherwise have been unwilling to participate in the study for fear of litigation. This sampling technique has the potential to introduce selection bias.

The Zimbabwean MoHCC has considered addressing the knowledge gap about PrEP in the general population through different channels whilst at the same time raising awareness to increase risk perception, especially amongst adolescent girls and young women. These considerations have been made as part of an implementation plan for PrEP in Zimbabwe between 2018 and 2020 (inclusive). Our study provides guidance on the progress made on addressing the knowledge gap about PrEP.

## Conclusion

We set out to evaluate the knowledge levels of PrEP and the likelihood of its use amongst FSWs. Whilst the knowledge level was low, the majority of FSWs would be willing to use PrEP for the purpose of HIV/AIDS prevention. Non-governmental organisations are playing a major role in sensitising FSWs about PrEP. The local clinics need to increase their visibility as information dissemination institutions for PrEP. The clinics are strategically positioned to enlighten key populations about PrEP and to prescribe medication to those who might need it. Successful dissemination of information can be achieved if PrEP programmes are incorporated into other programmes within the clinics.

## References

[1] Takarinda KC, Madyira LK, Mhangara M, et al Factors associated with ever being HIV-tested in Zimbabwe: An extended analysis of the Zimbabwe Demographic and Health Survey (2010–2011). PLoS One. 2016;11(1):e0147828 10.1371/journal.pone.0147828 26808547 PMC4726692

[2] Cowan FM, Mtetwa S, Davey C, et al Engagement with HIV prevention treatment and care among female sex workers in Zimbabwe: A respondent driven sampling survey. PLoS One. 2013;8(10):e77080 10.1371/journal.pone.0077080 24143203 PMC3797143

[3] Beyrer C, Baral SD, Collins C, et al The global response to HIV in men who have sex with men. Lancet. 2016;388(10040):198–206. 10.1016/S0140-6736(16)30781-4 27411880

[4] National Medicines and Therapeutics Policy Advisory Committee (NMTPAC) Guidelines for antiretroviral therapy for the prevention and treatment of HIV in Zimbabwe, Harare: Ministry of Health and Child Care; 2016.

[5] Joint United Nations Programme on HIV/AIDS (UNAIDS) 90-90-90 an ambitious treatment target to help end the AIDS epidemic, Geneva: UNAIDS; 2014.

[6] Desai M, Field N, Grant R, McCormack S State of the art review: Recent advances in PrEP for HIV. BMJ. 2017;359:j5011 10.1136/bmj.j5011 29229609 PMC6020995

[7] Cowan FM, Chabata ST, Musemburi S, et al Strengthening the scale- p and uptake of effective interventions for sex workers for population impact in Zimbabwe. J Int AIDS Soc. 2019;22 (Suppl 4):e25320 10.1002/jia2.25320 31328445 PMC6643097

[8] Chiyaka T, Mushati P, Hensen B, et al Reaching young women who sell sex: Methods and results of social mapping to describe and identify young women for DREAMS impact evaluation in Zimbabwe. PLoS One. 2018;13(3):e0194301 10.1371/journal.pone.0194301 29543858 PMC5854392

[9] Gwavava E Understanding the uptake and retention patterns of PrEP users in Zimbabwe by subpopulation. AIDS 2018. Amsterdam: International AIDS Society; 2018.

[10] Johnson JW, LeBreton JM History and use of relative importance indices in organizational research. Organ Res Methods. 2004;7(3):238–257. 10.1177/1094428104266510

[11] Brooks RA, Kaplan RL, Lieber E, Landovitz RJ, Lee SJ, Leibowitz AA Motivators, concerns, and barriers to adoption of preexposure prophylaxis for HIV prevention among gay and bisexual men in HIV-serodiscordant male relationships. AIDS Care. 2011;23(9):1136–1145. 10.1080/09540121.2011.554528 21476147 PMC3244470

[12] Peng B, Yang X, Zhang Y, et al Willingness to use pre-exposure prophylaxis for HIV prevention among female sex workers: A cross-sectional study in China. HIV AIDS. 2012;4:149–158. 10.2147/HIV.S33445 PMC3463398 23055781

[13] Reza-Paul S, Lazarus L, Doshi M, et al Prioritizing risk in preparation for a demonstration project: A mixed methods feasibility study of oral pre-exposure prophylaxis (PREP) among female sex workers in South India. PLoS One. 2016;11(11):e0166889 10.1371/journal.pone.0166889 27880833 PMC5120793

[14] Cohen SE, Vittinghoff E, Bacon O, et al High interest in pre-exposure prophylaxis among men who have sex with men at risk for HIV-infection: Baseline data from the US PrEP demonstration project. J Acquir Immune Defic Syndr. 2015;68(4):439–448. 10.1097/QAI.0000000000000479 25501614 PMC4334721

[15] Whetham J, Taylor S, Charlwood L, et al Pre-exposure prophylaxis for conception (PrEP-C) as a risk reduction strategy in HIV-positive men and HIV-negative women in the UK. AIDS Care. 2014;26(3):332–336. 10.1080/09540121.2013.819406 23876052

[16] Vernazza PL, Graf I, Sonnenberg-Schwan U, Geit M, Meurer A Preexposure prophylaxis and timed intercourse for HIV-discordant couples willing to conceive a child. AIDS. 2011;25(16):2005–2008. 10.1097/QAD.0b013e32834a36d0 21716070

[17] Dolling DI, Desai M, McOwan A, et al An analysis of baseline data from the PROUD study: An open-label randomised trial of pre-exposure prophylaxis. Trials. 2016;17:163 10.1186/s13063-016-1286-4 27013513 PMC4806447

[18] Elmes J, Nhongo K, Ward H, et al The price of sex: Condom use and the determinants of the price of sex among female sex workers in eastern Zimbabwe. J Infect Dis. 2014;210 (Suppl 2):S569–S578. 10.1093/infdis/jiu493 25381377 PMC4231645

[19] Wilton J, Senn H, Sharma M, Tan DH Pre-exposure prophylaxis for sexually-acquired HIV risk management: A review. HIV AIDS. 2015;7:125–136. 10.2147/HIV.S50025 PMC4422285 25987851

[20] Mack N, Odhiambo J, Wong CM, Agot K Barriers and facilitators to pre-exposure prophylaxis (PrEP) eligibility screening and ongoing HIV testing among target populations in Bondo and Rarieda, Kenya: Results of a consultation with community stakeholders. BMC Health Serv Res. 2014;14:231 10.1186/1472-6963-14-231 24886646 PMC4051373

[21] Heffron R, Celum C, Mugo N, et al., editors High initiation of PrEP and ART in a demonstration project among African HIV-discordant couples. 21st Conference on Retroviruses and Opportunistic Infections; 2014 March 03–06; Boston, MA: International Aids Society; 2014.

[22] Restar AJ, Tocco JU, Mantell JE, et al Perspectives on HIV pre- and post-exposure prophylaxes (PrEP and PEP) among female and male sex workers in Mombasa, Kenya: Implications for integrating biomedical prevention into sexual health services. AIDS Educ Prev. 2017;29(2):141–153. 10.1521/aeap.2017.29.2.141 28467163 PMC5706461

[23] Auerbach JD, Kinsky S, Brown G, Charles V Knowledge, attitudes, and likelihood of pre-exposure prophylaxis (PrEP) use among US women at risk of acquiring HIV. AIDS Patient Care STDS. 2015;29(2):102–110. 10.1089/apc.2014.0142 25513954 PMC4321978

